# [2,6-Di­fluoro-3-(pyridin-2-yl-κ*N*)pyridin-4-yl-κ*C* ^4^](penta­ne-2,4-dionato-κ^2^ *O*,*O*′)iridium(III)

**DOI:** 10.1107/S1600536813026160

**Published:** 2013-10-09

**Authors:** Kaijun Luo, Chenyang Zhang, Juan Jia, Daibing Luo

**Affiliations:** a College of Chemistry and Materials Science, Sichuan Normal University, Chengdu, Sichuan 610068, People’s Republic of China; b Analytical and Testing Center, Sichuan University, Chengdu, Sichuan 610065, People’s Republic of China

## Abstract

The title compound, [Ir(C_10_H_5_F_2_N_2_)_2_(C_5_H_7_O_2_)], has a distorted octa­hedral coordination geometry around the Ir^III^ atom, retaining the *cis*-C,*C*/*trans*-N,*N* chelate disposition in two 2,6-di­fluoro-3-(pyridin-2-yl-κ*N*)pyridin-4-yl ligands which are nearly mutually perpendicular [dihedral angle = 82.75 (15)°]. The mol­ecular structure is stabilized by weak C—H⋯O and C—H⋯F hydrogen-bond inter­actions. The crystal structure is stabilized by π–π stacking inter­actions (centroid–centroid distance = 3.951 Å).

## Related literature

For general background and related structures, see: Xiao *et al.* (2011 ▶); Lamansky *et al.* (2001*a* ▶); Lee *et al.* (2009 ▶); Jung *et al.* (2012 ▶). For the synthesis of the title complex, see: Lamansky *et al.* (2001*b* ▶); Luo *et al.* (2011 ▶).
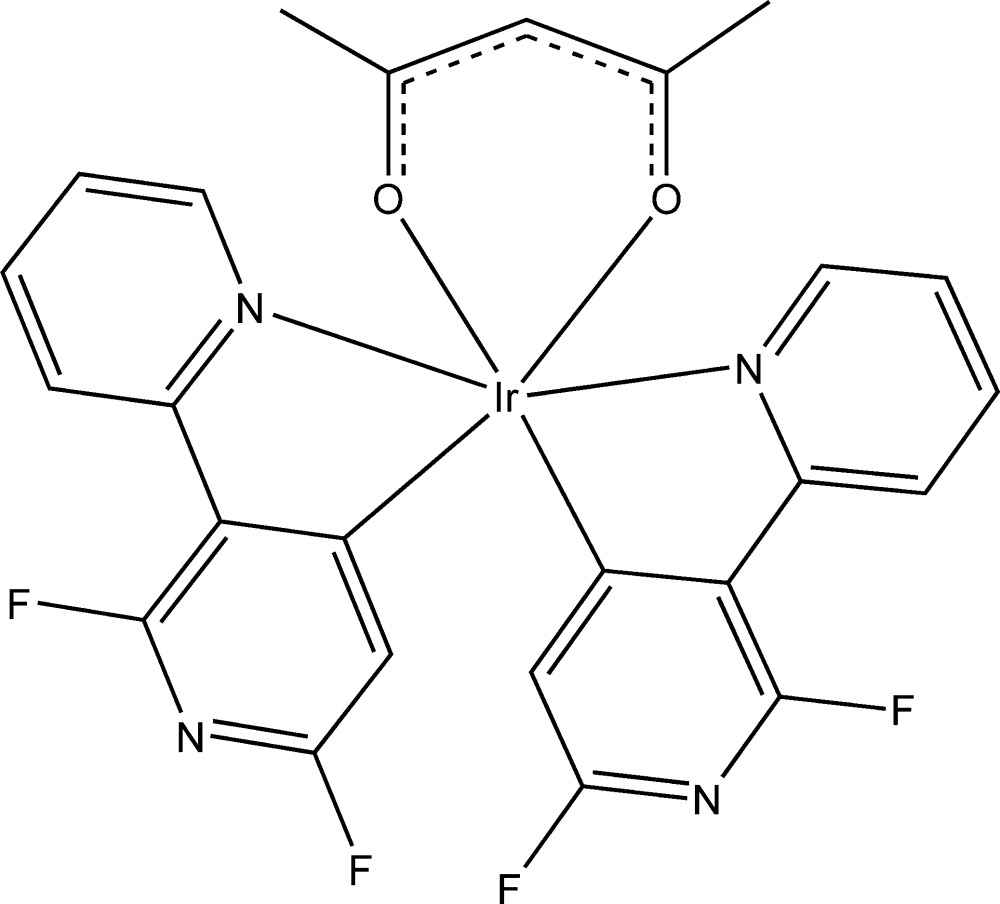


## Experimental

### 

#### Crystal data

[Ir(C_10_H_5_F_2_N_2_)_2_(C_5_H_7_O_2_)]
*M* *_r_* = 673.63
Monoclinic, 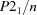

*a* = 7.7126 (2) Å
*b* = 18.2039 (5) Å
*c* = 16.3534 (4) Å
β = 99.371 (3)°
*V* = 2265.38 (10) Å^3^
*Z* = 4
Mo *K*α radiation
μ = 5.96 mm^−1^
*T* = 293 K
0.40 × 0.35 × 0.35 mm

#### Data collection

Agilent Xcalibur Eos diffractometer
Absorption correction: multi-scan (*CrysAlis PRO*; Agilent, 2011 ▶) *T*_min_ = 0.400, *T*_max_ = 1.000
9371 measured reflections
4628 independent reflections
3736 reflections with *I* > 2σ(*I*)
*R*_int_ = 0.030

#### Refinement

*R*[*F* ^2^ > 2σ(*F* ^2^)] = 0.032
*wR*(*F* ^2^) = 0.066
*S* = 1.03
4628 reflections
327 parameters
H-atom parameters constrained
Δρ_max_ = 1.17 e Å^−3^
Δρ_min_ = −0.97 e Å^−3^

### 

Data collection: *CrysAlis PRO* (Agilent, 2011 ▶); cell refinement: *CrysAlis PRO*; data reduction: *CrysAlis PRO*; program(s) used to solve structure: *SHELXS97* (Sheldrick, 2008 ▶); program(s) used to refine structure: *SHELXL97* (Sheldrick, 2008 ▶); molecular graphics: *OLEX2* (Dolomanov *et al.*, 2009 ▶); software used to prepare material for publication: *OLEX2*.

## Supplementary Material

Crystal structure: contains datablock(s) global, I. DOI: 10.1107/S1600536813026160/bx2449sup1.cif

Structure factors: contains datablock(s) I. DOI: 10.1107/S1600536813026160/bx2449Isup2.hkl

Additional supplementary materials:  crystallographic information; 3D view; checkCIF report

## Figures and Tables

**Table 1:** Hydrogen-bond geometry (Å, °)

*D*—H⋯*A*	*D*—H	H⋯*A*	*D*⋯*A*	*D*—H⋯*A*
C1—H1⋯O2	0.93	2.56	3.146 (6)	121
C4—H4⋯F2	0.93	2.36	2.894 (7)	121
C11—H11⋯O1	0.93	2.59	3.175 (7)	121
C14—H14⋯F4	0.93	2.33	2.915 (7)	121

**Fig. 1.**
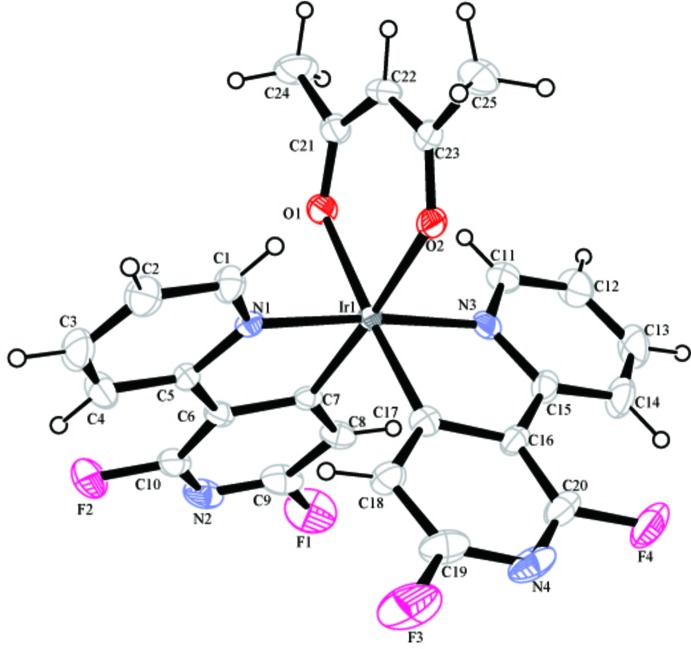
The molecular structure of the title complex, with atom labels and 50% probability displacement ellipsoids.

## supplementary crystallographic information

### 1. Comment

The phosphorescent cyclometalated Iridium(III) complexes have received considerable attention in the fabrication of phosphorescent organic light emitting diodes for their high quantum efficiencies, relatively short phosphorescent lifetimes. Lee *et al.* (2009) reported a *fac*-Ir(III) complex, *fac*-Ir(dfpypy)_3_, using 2',6'-Difluoro-2,3'-bipyridine (dfpypy)ligand, this complex exhibits intense blue emission with high color purity. Recently, a heteroleptic Ir complex,(dfpypy)_2_Ir(acac), incorporating dfpypy and acetyl acetone, was synthesized by our group and the blue phosphorescent electroluminescence devices were fabricated by doped (dfpypy)_2_Ir(acac) into PVK host. We report herein on its crystal structure.

The structure of the title complex is shown in Fig. 1. The coordination at the iridium atom is octahedral. The title compound displays shorter Ir–C [1.968 (5) and 1.975 (6) Å] and Ir–N [2.036 (4) and 2.047 (4) Å] bond distances, compared with those in *fac*-Ir(dfpypy)_3_ [1.997 (5)\sim 2.005 (5) Å for Ir–C bands and 2.116 (4)~2.135 (4) Å for Ir–N bands]. Ir–O bands are 2.143 (3) and 2.147 (3), respectively.

The interplanar angles between the chelate rings, for IrO_2_C_3_ ring A (Ir1, O2, C23, C22, C21, O1) to the IrNC_3_ ring B (Ir1, N1, C5, C6, C7) and C (Ir1, N3, C15, C16, C17) are 89.37 and 87.22 °, respectively, while the IrNC_3_ rings, B and C, are inclined at an angle of 86.71 °. The dihedral angles between two pyridyl rings of fluorine-substitution bipyridine ligands are 6.94 ° for bipyridine involving N1, N2 atoms and 8.24 ° for that involving N3, N4 atoms, which has bigger dihedral angles than those in *fac*-Ir(dfpypy)_3_ [3.84~ 5.63]. The molecular structure is stabilized by weak C–H···O and C–H···F hydrogen bonds interactions, Table 1. The crystal structure is stabilized by π-π stacking interactions(Cg1- Cg2 distance 3.951Å, Cg1=N1-C1/C5 ; Cg2= N3-C11/C15)

### 2. Experimental

2',6'-Difluoro-2,3'-bipyridine (dfpypy) ligand was prepared according to the method of Lee *et al.* (2009). The Ir(III) µ-dichloro-bridged dimer, [IrCl(dfpypy)_2_]_2_ was prepared according to the method of Lamansky *et al.* (2001a). A mixture of [IrCl(dfpypy)_2_]_2_ (0.82 mmol, 1.04 g), acetyl acetone (4.2 mmol, 0.42 g), anhydrous Cs_2_CO_3_ (0.8 mmol. 0.265 g) and 2-ethoxyethanol (30 ml) was stirred under inert atmosphere at 95 °C for 15 h. After cooling to room temperature, the precipitate was fitered off, washed with water and hexane and methanol. The crude product was purified by chromatography on silica gel [eluent: petroleum ether and ethyl acetate, *v*/*v* = 3:1]. The greenish yellow crystals, suitable for X-ray analysis, were obtained by slow diffusion of methanol into the dichloromethane solution of the title complex.

### 3. Refinement

H atoms were placed at calculated positions and refined as riding atoms: C–H = 0.93, 0.96 Å for CH (aromatic) and CH_3_ H-atoms, respectively, with *U*_iso_(H) = k × *U*_eq_(C), where k = 1.5 for CH_3_ H atoms, and k = 1.2 for all other H atoms.

### Crystal data

**Table:**

[Ir(C_10_H_5_F_2_N_2_)_2_(C_5_H_7_O_2_)]	*F*(000) = 1296
*M**_r_* = 673.63	*D*_x_ = 1.975 Mg m^−^^3^
Monoclinic, *P*2_1_/*n*	Mo *K*α radiation, λ = 0.7107 Å
Hall symbol: -P 2yn	Cell parameters from 3878 reflections
*a* = 7.7126 (2) Å	θ = 3.0–29.1°
*b* = 18.2039 (5) Å	µ = 5.96 mm^−^^1^
*c* = 16.3534 (4) Å	*T* = 293 K
β = 99.371 (3)°	Block, yellow
*V* = 2265.38 (10) Å^3^	0.40 × 0.35 × 0.35 mm
*Z* = 4	

### Data collection

**Table:**

Agilent Xcalibur Eos diffractometer	4628 independent reflections
Radiation source: Enhance (Mo) X-ray Source	3736 reflections with *I* > 2σ(*I*)
Graphite monochromator	*R*_int_ = 0.030
Detector resolution: 16.0874 pixels mm^-1^	θ_max_ = 26.4°, θ_min_ = 3.0°
ω scans	*h* = −9→9
Absorption correction: multi-scan (*CrysAlis PRO*; Agilent, 2011)	*k* = −19→22
*T*_min_ = 0.400, *T*_max_ = 1.000	*l* = −11→20
9371 measured reflections	

### Refinement

**Table:**

Refinement on *F*^2^	0 restraints
Least-squares matrix: full	H-atom parameters constrained
*R*[*F*^2^ > 2σ(*F*^2^)] = 0.032	*w* = 1/[σ^2^(*F*_o_^2^) + (0.0236*P*)^2^] where *P* = (*F*_o_^2^ + 2*F*_c_^2^)/3
*wR*(*F*^2^) = 0.066	(Δ/σ)_max_ = 0.005
*S* = 1.03	Δρ_max_ = 1.17 e Å^−^^3^
4628 reflections	Δρ_min_ = −0.97 e Å^−^^3^
327 parameters	

### Special details

**Table:**

Experimental. Absorption correction: CrysAlisPro, Agilent Technologies, Version 1.171.35.11 (release 16-05-2011 CrysAlis171 .NET) (compiled May 16 2011,17:55:39) Empirical absorption correction using spherical harmonics, implemented in SCALE3 ABSPACK scaling algorithm.
Geometry. All e.s.d.'s (except the e.s.d. in the dihedral angle between two l.s. planes) are estimated using the full covariance matrix. The cell e.s.d.'s are taken into account individually in the estimation of e.s.d.'s in distances, angles and torsion angles; correlations between e.s.d.'s in cell parameters are only used when they are defined by crystal symmetry. An approximate (isotropic) treatment of cell e.s.d.'s is used for estimating e.s.d.'s involving l.s. planes.
Refinement. Refinement of *F*^2^ against ALL reflections. The weighted *R*-factor *wR* and goodness of fit *S* are based on *F*^2^, conventional *R*-factors *R* are based on *F*, with *F* set to zero for negative *F*^2^. The threshold expression of *F*^2^ > σ(*F*^2^) is used only for calculating *R*-factors(gt) *etc*. and is not relevant to the choice of reflections for refinement. *R*-factors based on *F*^2^ are statistically about twice as large as those based on *F*, and *R*- factors based on ALL data will be even larger.

### Fractional atomic coordinates and isotropic or equivalent isotropic displacement parameters (Å^2^)

**Table:**

	*x*	*y*	*z*	*U*_iso_*/*U*_eq_	
Ir1	0.77717 (2)	0.232240 (10)	0.507721 (11)	0.02588 (7)	
F1	0.3566 (5)	0.0373 (2)	0.6253 (3)	0.0892 (13)	
F2	0.8659 (5)	0.08762 (19)	0.78652 (19)	0.0737 (11)	
F3	1.2168 (5)	0.0322 (2)	0.4142 (3)	0.0963 (14)	
F4	0.7182 (5)	0.0727 (2)	0.2402 (2)	0.0750 (12)	
O1	0.6653 (4)	0.31813 (19)	0.57247 (19)	0.0374 (8)	
O2	0.8806 (4)	0.31507 (19)	0.43594 (19)	0.0374 (8)	
N1	0.9910 (5)	0.2314 (2)	0.5990 (2)	0.0286 (9)	
N2	0.6126 (7)	0.0637 (3)	0.7057 (3)	0.0544 (14)	
N3	0.5654 (5)	0.2222 (2)	0.4151 (3)	0.0350 (10)	
N4	0.9680 (7)	0.0529 (3)	0.3283 (3)	0.0593 (15)	
C1	1.1398 (7)	0.2682 (3)	0.5962 (3)	0.0410 (13)	
H1	1.1449	0.2997	0.5519	0.049*	
C2	1.2847 (8)	0.2615 (3)	0.6560 (4)	0.0534 (16)	
H2	1.3871	0.2872	0.6516	0.064*	
C3	1.2778 (8)	0.2170 (4)	0.7220 (4)	0.0607 (19)	
H3	1.3744	0.2128	0.7639	0.073*	
C4	1.1265 (8)	0.1783 (3)	0.7263 (3)	0.0547 (16)	
H4	1.1214	0.1473	0.7710	0.066*	
C5	0.9813 (6)	0.1851 (3)	0.6641 (3)	0.0332 (12)	
C6	0.8145 (7)	0.1469 (3)	0.6568 (3)	0.0341 (12)	
C7	0.6973 (6)	0.1582 (3)	0.5817 (3)	0.0296 (11)	
C8	0.5390 (6)	0.1191 (3)	0.5712 (3)	0.0413 (13)	
H8	0.4567	0.1232	0.5231	0.050*	
C9	0.5109 (8)	0.0743 (3)	0.6354 (4)	0.0533 (16)	
C10	0.7617 (8)	0.0994 (3)	0.7133 (3)	0.0468 (15)	
C11	0.4124 (7)	0.2577 (3)	0.4142 (4)	0.0451 (14)	
H11	0.4027	0.2911	0.4562	0.054*	
C12	0.2703 (8)	0.2464 (3)	0.3536 (4)	0.0582 (18)	
H12	0.1652	0.2711	0.3547	0.070*	
C13	0.2858 (8)	0.1980 (4)	0.2915 (4)	0.0664 (19)	
H13	0.1918	0.1902	0.2490	0.080*	
C14	0.4404 (8)	0.1612 (4)	0.2920 (3)	0.0607 (18)	
H14	0.4501	0.1274	0.2503	0.073*	
C15	0.5835 (7)	0.1735 (3)	0.3542 (3)	0.0391 (13)	
C16	0.7538 (7)	0.1371 (3)	0.3668 (3)	0.0359 (12)	
C17	0.8659 (6)	0.1543 (3)	0.4420 (3)	0.0334 (12)	
C18	1.0248 (7)	0.1163 (3)	0.4584 (3)	0.0428 (14)	
H18	1.1017	0.1235	0.5077	0.051*	
C19	1.0645 (8)	0.0681 (3)	0.3999 (4)	0.0576 (18)	
C20	0.8178 (8)	0.0878 (3)	0.3146 (3)	0.0500 (16)	
C21	0.6764 (7)	0.3863 (3)	0.5576 (3)	0.0411 (13)	
C22	0.7633 (7)	0.4182 (3)	0.4978 (3)	0.0482 (15)	
H22	0.7578	0.4692	0.4944	0.058*	
C23	0.8565 (7)	0.3840 (3)	0.4426 (3)	0.0405 (13)	
C24	0.5894 (9)	0.4356 (3)	0.6109 (4)	0.070 (2)	
H24A	0.4649	0.4274	0.6003	0.105*	
H24B	0.6135	0.4858	0.5987	0.105*	
H24C	0.6337	0.4256	0.6681	0.105*	
C25	0.9410 (8)	0.4313 (3)	0.3859 (4)	0.0654 (19)	
H25A	1.0634	0.4191	0.3911	0.098*	
H25B	0.9291	0.4820	0.4002	0.098*	
H25C	0.8850	0.4233	0.3297	0.098*	

### Atomic displacement parameters (Å^2^)

**Table:**

	*U*^11^	*U*^22^	*U*^33^	*U*^12^	*U*^13^	*U*^23^
Ir1	0.02867 (11)	0.02454 (12)	0.02474 (11)	−0.00052 (8)	0.00525 (8)	−0.00004 (9)
F1	0.077 (3)	0.079 (3)	0.120 (4)	−0.034 (2)	0.040 (2)	0.024 (3)
F2	0.117 (3)	0.062 (3)	0.041 (2)	0.004 (2)	0.011 (2)	0.0232 (18)
F3	0.069 (3)	0.089 (3)	0.137 (4)	0.028 (2)	0.033 (3)	−0.043 (3)
F4	0.104 (3)	0.074 (3)	0.049 (2)	−0.017 (2)	0.017 (2)	−0.0316 (19)
O1	0.045 (2)	0.036 (2)	0.0335 (19)	0.0060 (17)	0.0115 (16)	0.0006 (17)
O2	0.051 (2)	0.034 (2)	0.0289 (18)	−0.0077 (17)	0.0102 (16)	−0.0006 (17)
N1	0.031 (2)	0.025 (2)	0.030 (2)	0.0012 (18)	0.0060 (18)	−0.0049 (19)
N2	0.081 (4)	0.037 (3)	0.053 (3)	0.006 (3)	0.036 (3)	0.013 (3)
N3	0.041 (3)	0.030 (3)	0.033 (2)	−0.0038 (19)	0.004 (2)	0.008 (2)
N4	0.072 (4)	0.048 (3)	0.067 (4)	−0.009 (3)	0.041 (3)	−0.024 (3)
C1	0.034 (3)	0.049 (4)	0.039 (3)	−0.008 (3)	0.001 (3)	−0.002 (3)
C2	0.045 (4)	0.055 (4)	0.057 (4)	−0.005 (3)	0.000 (3)	−0.003 (3)
C3	0.050 (4)	0.068 (5)	0.055 (4)	0.012 (3)	−0.017 (3)	−0.013 (4)
C4	0.064 (4)	0.053 (4)	0.044 (3)	0.014 (3)	−0.002 (3)	0.010 (3)
C5	0.041 (3)	0.030 (3)	0.028 (3)	0.006 (2)	0.002 (2)	−0.002 (2)
C6	0.047 (3)	0.026 (3)	0.031 (3)	0.005 (2)	0.014 (2)	0.004 (2)
C7	0.038 (3)	0.024 (3)	0.031 (3)	0.003 (2)	0.015 (2)	−0.001 (2)
C8	0.038 (3)	0.033 (3)	0.055 (4)	0.000 (2)	0.015 (3)	0.006 (3)
C9	0.059 (4)	0.038 (4)	0.072 (5)	−0.008 (3)	0.036 (4)	0.010 (3)
C10	0.076 (4)	0.034 (3)	0.035 (3)	0.007 (3)	0.022 (3)	0.004 (3)
C11	0.043 (3)	0.040 (4)	0.052 (4)	0.006 (3)	0.007 (3)	0.004 (3)
C12	0.037 (3)	0.063 (5)	0.068 (5)	−0.002 (3)	−0.012 (3)	0.005 (4)
C13	0.055 (4)	0.079 (5)	0.053 (4)	−0.016 (4)	−0.026 (3)	0.005 (4)
C14	0.067 (4)	0.069 (5)	0.040 (3)	−0.022 (4)	−0.010 (3)	−0.010 (3)
C15	0.048 (3)	0.041 (3)	0.029 (3)	−0.011 (3)	0.007 (3)	−0.001 (3)
C16	0.046 (3)	0.033 (3)	0.030 (3)	−0.008 (2)	0.011 (2)	−0.006 (2)
C17	0.033 (3)	0.033 (3)	0.037 (3)	−0.007 (2)	0.012 (2)	0.002 (2)
C18	0.044 (3)	0.041 (4)	0.044 (3)	0.004 (3)	0.010 (3)	−0.009 (3)
C19	0.056 (4)	0.044 (4)	0.082 (5)	0.005 (3)	0.036 (4)	−0.010 (4)
C20	0.072 (4)	0.042 (4)	0.041 (3)	−0.022 (3)	0.023 (3)	−0.012 (3)
C21	0.052 (3)	0.037 (4)	0.035 (3)	0.009 (3)	0.009 (3)	0.000 (3)
C22	0.072 (4)	0.025 (3)	0.048 (4)	0.003 (3)	0.010 (3)	0.002 (3)
C23	0.053 (3)	0.035 (3)	0.031 (3)	−0.008 (3)	0.000 (3)	−0.002 (3)
C24	0.110 (6)	0.038 (4)	0.068 (5)	0.020 (4)	0.034 (4)	−0.010 (3)
C25	0.101 (5)	0.045 (4)	0.054 (4)	−0.021 (4)	0.025 (4)	0.012 (3)

### Geometric parameters (Å, º)

**Table:**

Ir1—O1	2.147 (3)	C6—C10	1.374 (7)
Ir1—O2	2.143 (3)	C7—C8	1.400 (6)
Ir1—N1	2.036 (4)	C8—H8	0.9300
Ir1—N3	2.047 (4)	C8—C9	1.375 (7)
Ir1—C7	1.975 (5)	C11—H11	0.9300
Ir1—C17	1.968 (5)	C11—C12	1.368 (8)
F1—C9	1.353 (6)	C12—H12	0.9300
F2—C10	1.346 (6)	C12—C13	1.363 (9)
F3—C19	1.331 (6)	C13—H13	0.9300
F4—C20	1.358 (6)	C13—C14	1.367 (8)
O1—C21	1.270 (6)	C14—H14	0.9300
O2—C23	1.276 (6)	C14—C15	1.391 (7)
N1—C1	1.336 (6)	C15—C16	1.456 (7)
N1—C5	1.369 (6)	C16—C17	1.419 (7)
N2—C9	1.295 (7)	C16—C20	1.383 (7)
N2—C10	1.309 (7)	C17—C18	1.395 (6)
N3—C11	1.343 (6)	C18—H18	0.9300
N3—C15	1.357 (6)	C18—C19	1.368 (7)
N4—C19	1.311 (8)	C21—C22	1.400 (7)
N4—C20	1.309 (7)	C21—C24	1.485 (7)
C1—H1	0.9300	C22—H22	0.9300
C1—C2	1.365 (7)	C22—C23	1.389 (7)
C2—H2	0.9300	C23—C25	1.491 (7)
C2—C3	1.357 (8)	C24—H24A	0.9600
C3—H3	0.9300	C24—H24B	0.9600
C3—C4	1.375 (8)	C24—H24C	0.9600
C4—H4	0.9300	C25—H25A	0.9600
C4—C5	1.391 (7)	C25—H25B	0.9600
C5—C6	1.451 (6)	C25—H25C	0.9600
C6—C7	1.417 (6)		
			
O2—Ir1—O1	88.46 (14)	N2—C10—F2	113.2 (5)
N1—Ir1—O1	89.22 (13)	N2—C10—C6	126.9 (6)
N1—Ir1—O2	94.42 (14)	N3—C11—H11	118.8
N1—Ir1—N3	174.41 (16)	N3—C11—C12	122.3 (6)
N3—Ir1—O1	95.37 (15)	C12—C11—H11	118.8
N3—Ir1—O2	88.90 (14)	C11—C12—H12	120.7
C7—Ir1—O1	90.23 (16)	C13—C12—C11	118.6 (6)
C7—Ir1—O2	175.19 (16)	C13—C12—H12	120.7
C7—Ir1—N1	80.93 (17)	C12—C13—H13	120.2
C7—Ir1—N3	95.83 (17)	C12—C13—C14	119.6 (6)
C17—Ir1—O1	176.01 (16)	C14—C13—H13	120.2
C17—Ir1—O2	90.81 (16)	C13—C14—H14	119.6
C17—Ir1—N1	94.75 (18)	C13—C14—C15	120.8 (6)
C17—Ir1—N3	80.69 (18)	C15—C14—H14	119.6
C17—Ir1—C7	90.8 (2)	N3—C15—C14	118.5 (5)
C21—O1—Ir1	124.9 (3)	N3—C15—C16	113.1 (4)
C23—O2—Ir1	124.9 (3)	C14—C15—C16	128.3 (5)
C1—N1—Ir1	124.7 (4)	C17—C16—C15	115.5 (5)
C1—N1—C5	119.4 (4)	C20—C16—C15	127.8 (5)
C5—N1—Ir1	115.7 (3)	C20—C16—C17	116.8 (5)
C9—N2—C10	114.0 (5)	C16—C17—Ir1	114.6 (4)
C11—N3—Ir1	124.0 (4)	C18—C17—Ir1	128.7 (4)
C11—N3—C15	120.1 (5)	C18—C17—C16	116.7 (5)
C15—N3—Ir1	115.9 (3)	C17—C18—H18	120.9
C20—N4—C19	114.1 (5)	C19—C18—C17	118.3 (5)
N1—C1—H1	118.7	C19—C18—H18	120.9
N1—C1—C2	122.6 (5)	F3—C19—C18	118.9 (6)
C2—C1—H1	118.7	N4—C19—F3	114.1 (6)
C1—C2—H2	120.4	N4—C19—C18	126.9 (6)
C3—C2—C1	119.2 (6)	F4—C20—C16	118.6 (6)
C3—C2—H2	120.4	N4—C20—F4	114.2 (5)
C2—C3—H3	120.3	N4—C20—C16	127.2 (6)
C2—C3—C4	119.4 (6)	O1—C21—C22	126.5 (5)
C4—C3—H3	120.3	O1—C21—C24	115.3 (5)
C3—C4—H4	119.8	C22—C21—C24	118.2 (5)
C3—C4—C5	120.4 (6)	C21—C22—H22	115.7
C5—C4—H4	119.8	C23—C22—C21	128.7 (5)
N1—C5—C4	119.0 (5)	C23—C22—H22	115.7
N1—C5—C6	113.1 (4)	O2—C23—C22	126.5 (5)
C4—C5—C6	127.9 (5)	O2—C23—C25	115.5 (5)
C7—C6—C5	115.7 (4)	C22—C23—C25	118.0 (5)
C10—C6—C5	127.1 (5)	C21—C24—H24A	109.5
C10—C6—C7	117.2 (5)	C21—C24—H24B	109.5
C6—C7—Ir1	114.3 (3)	C21—C24—H24C	109.5
C8—C7—Ir1	128.7 (4)	H24A—C24—H24B	109.5
C8—C7—C6	116.9 (5)	H24A—C24—H24C	109.5
C7—C8—H8	121.6	H24B—C24—H24C	109.5
C9—C8—C7	116.8 (5)	C23—C25—H25A	109.5
C9—C8—H8	121.6	C23—C25—H25B	109.5
F1—C9—C8	116.5 (6)	C23—C25—H25C	109.5
N2—C9—F1	115.4 (6)	H25A—C25—H25B	109.5
N2—C9—C8	128.1 (6)	H25A—C25—H25C	109.5
F2—C10—C6	119.9 (6)	H25B—C25—H25C	109.5
			
Ir1—O1—C21—C22	−0.5 (8)	C4—C5—C6—C10	4.9 (9)
Ir1—O1—C21—C24	−179.6 (4)	C5—N1—C1—C2	0.2 (8)
Ir1—O2—C23—C22	1.3 (7)	C5—C6—C7—Ir1	−6.0 (5)
Ir1—O2—C23—C25	−179.8 (3)	C5—C6—C7—C8	176.9 (4)
Ir1—N1—C1—C2	−174.7 (4)	C5—C6—C10—F2	3.9 (8)
Ir1—N1—C5—C4	176.1 (4)	C5—C6—C10—N2	−178.5 (5)
Ir1—N1—C5—C6	−2.2 (5)	C6—C7—C8—C9	1.1 (7)
Ir1—N3—C11—C12	−176.1 (4)	C7—Ir1—O1—C21	176.7 (4)
Ir1—N3—C15—C14	176.3 (4)	C7—Ir1—O2—C23	−76.0 (19)
Ir1—N3—C15—C16	0.0 (5)	C7—Ir1—N1—C1	174.4 (4)
Ir1—C7—C8—C9	−175.5 (4)	C7—Ir1—N1—C5	−0.8 (3)
Ir1—C17—C18—C19	−174.8 (4)	C7—Ir1—N3—C11	84.2 (4)
O1—Ir1—O2—C23	−1.7 (4)	C7—Ir1—N3—C15	−92.5 (4)
O1—Ir1—N1—C1	−95.3 (4)	C7—Ir1—C17—C16	100.6 (4)
O1—Ir1—N1—C5	89.6 (3)	C7—Ir1—C17—C18	−81.6 (5)
O1—Ir1—N3—C11	−6.6 (4)	C7—C6—C10—F2	−178.5 (4)
O1—Ir1—N3—C15	176.7 (3)	C7—C6—C10—N2	−0.9 (8)
O1—Ir1—C7—C6	−85.5 (3)	C7—C8—C9—F1	179.2 (5)
O1—Ir1—C7—C8	91.2 (4)	C7—C8—C9—N2	0.6 (9)
O1—Ir1—C17—C16	−4 (2)	C9—N2—C10—F2	−179.8 (5)
O1—Ir1—C17—C18	173 (2)	C9—N2—C10—C6	2.4 (9)
O1—C21—C22—C23	−0.7 (9)	C10—N2—C9—F1	179.1 (5)
O2—Ir1—O1—C21	1.4 (4)	C10—N2—C9—C8	−2.3 (9)
O2—Ir1—N1—C1	−6.9 (4)	C10—C6—C7—Ir1	176.1 (4)
O2—Ir1—N1—C5	178.0 (3)	C10—C6—C7—C8	−1.0 (7)
O2—Ir1—N3—C11	−94.9 (4)	C11—N3—C15—C14	−0.6 (7)
O2—Ir1—N3—C15	88.3 (3)	C11—N3—C15—C16	−176.9 (4)
O2—Ir1—C7—C6	−11 (2)	C11—C12—C13—C14	1.3 (10)
O2—Ir1—C7—C8	165.3 (16)	C12—C13—C14—C15	−1.4 (10)
O2—Ir1—C17—C16	−83.9 (4)	C13—C14—C15—N3	1.0 (9)
O2—Ir1—C17—C18	93.8 (5)	C13—C14—C15—C16	176.8 (6)
N1—Ir1—O1—C21	95.8 (4)	C14—C15—C16—C17	−171.8 (5)
N1—Ir1—O2—C23	−90.8 (4)	C14—C15—C16—C20	7.8 (9)
N1—Ir1—N3—C11	138.5 (14)	C15—N3—C11—C12	0.5 (8)
N1—Ir1—N3—C15	−38.2 (16)	C15—C16—C17—Ir1	−6.4 (6)
N1—Ir1—C7—C6	3.7 (3)	C15—C16—C17—C18	175.6 (4)
N1—Ir1—C7—C8	−179.6 (5)	C15—C16—C20—F4	3.6 (8)
N1—Ir1—C17—C16	−178.4 (4)	C15—C16—C20—N4	−176.7 (5)
N1—Ir1—C17—C18	−0.7 (5)	C16—C17—C18—C19	2.9 (7)
N1—C1—C2—C3	−1.4 (9)	C17—Ir1—O1—C21	−78 (2)
N1—C5—C6—C7	5.3 (6)	C17—Ir1—O2—C23	174.3 (4)
N1—C5—C6—C10	−177.1 (5)	C17—Ir1—N1—C1	84.3 (4)
N3—Ir1—O1—C21	−87.4 (4)	C17—Ir1—N1—C5	−90.8 (3)
N3—Ir1—O2—C23	93.7 (4)	C17—Ir1—N3—C11	174.1 (4)
N3—Ir1—N1—C1	119.5 (14)	C17—Ir1—N3—C15	−2.7 (3)
N3—Ir1—N1—C5	−55.7 (15)	C17—Ir1—C7—C6	98.4 (4)
N3—Ir1—C7—C6	179.1 (3)	C17—Ir1—C7—C8	−85.0 (5)
N3—Ir1—C7—C8	−4.2 (5)	C17—C16—C20—F4	−176.7 (4)
N3—Ir1—C17—C16	4.8 (3)	C17—C16—C20—N4	3.0 (9)
N3—Ir1—C17—C18	−177.4 (5)	C17—C18—C19—F3	179.0 (5)
N3—C11—C12—C13	−0.9 (9)	C17—C18—C19—N4	−0.2 (9)
N3—C15—C16—C17	4.1 (6)	C19—N4—C20—F4	179.4 (5)
N3—C15—C16—C20	−176.3 (5)	C19—N4—C20—C16	−0.3 (9)
C1—N1—C5—C4	0.7 (7)	C20—N4—C19—F3	179.5 (5)
C1—N1—C5—C6	−177.6 (4)	C20—N4—C19—C18	−1.2 (9)
C1—C2—C3—C4	1.5 (9)	C20—C16—C17—Ir1	174.0 (4)
C2—C3—C4—C5	−0.7 (9)	C20—C16—C17—C18	−4.1 (7)
C3—C4—C5—N1	−0.4 (8)	C21—C22—C23—O2	0.3 (9)
C3—C4—C5—C6	177.5 (5)	C21—C22—C23—C25	−178.6 (5)
C4—C5—C6—C7	−172.8 (5)	C24—C21—C22—C23	178.4 (5)

### Hydrogen-bond geometry (Å, º)

**Table:**

*D*—H···*A*	*D*—H	H···*A*	*D*···*A*	*D*—H···*A*
C1—H1···O2	0.93	2.56	3.146 (6)	121
C4—H4···F2	0.93	2.36	2.894 (7)	121
C11—H11···O1	0.93	2.59	3.175 (7)	121
C14—H14···F4	0.93	2.33	2.915 (7)	121
